# Investigation of Guar Gum and Xanthan Gum Influence on Essential Thyme Oil Emulsion Properties and Encapsulation Release Using Modeling Tools

**DOI:** 10.3390/foods13060816

**Published:** 2024-03-07

**Authors:** Samara Ribeiro, Renata Almeida, Leonardo Batista, Janaina Lima, Ana Sarinho, Amanda Nascimento, Hugo Lisboa

**Affiliations:** Unidade Académica Engenharia de Alimentos, Universidade Federal Campina Grande, Av. Aprigio Veloso 882, Campina Grande 58400-900, Paraiba, Brazil

**Keywords:** hydrocolloid, release kinetics, emulsion, encapsulation

## Abstract

This study explores the influence of hydrocolloid interactions between Guar Gum (GG) and Xanthan Gum (XG) on the stability and release dynamics of essential thyme oil emulsions. We systematically characterized six emulsions with varying GG and XG ratios, employing spray-drying techniques for the encapsulation process. The stability of the emulsions was quantitatively analyzed, revealing a marked decrease in stability rates correlated with higher initial emulsion activity (zero-order kinetic constant r = −0.972). Furthermore, this study demonstrated that emulsions with carefully optimized hydrocolloid ratios could achieve high encapsulation efficiency (74%) and controlled release profiles. Kinetic modeling and diffusion analyses elucidated that increased XG concentrations tend to reduce diffusivity, thereby enhancing emulsion stability. The effective diffusivity of the thyme oil within the emulsion matrix was determined to be within a range of 0.7 to 2.4 × 10^−10^ m^2^/s, significantly influencing release kinetics. The Pearson correlation matrix underlined a substantial negative association between emulsion activity and effective diffusivity (r = −0.740), indicating that denser hydrocolloid networks impede oil mobility. The findings conclusively establish that the interplay of GG and XG concentrations is pivotal in dictating the emulsion’s physicochemical properties, with denser networks formed by higher XG content leading to slower oil release rates and enhanced stability. This research provides critical insights for the design of encapsulated food and pharmaceutical products, highlighting the imperative of strategic hydrocolloid selection to realize specific functional attributes and performance criteria.

## 1. Introduction

The encapsulation of volatile compounds, particularly essential oils, has garnered substantial interest in various fields, including food technology, pharmaceuticals, and cosmetics [1]. Essential oils, known for their potent biological activities and sensory attributes, present challenges in formulation due to their volatility and sensitivity to environmental factors, such as oxygen, light, and temperature [2]. The encapsulation technology offers a promising approach to protect these volatile compounds, enhancing their stability, controlled release, and integration into diverse product matrices [3].

Thyme oil, esteemed for its antimicrobial, antioxidant, and therapeutic properties, plays a pivotal role across the food, pharmaceutical, and cosmetic industries [4]. Extracted from the Thymus species, it is particularly valued for its phenolic compounds like thymol and carvacrol, which confer natural preservation capabilities against a wide range of microorganisms, enhancing food safety and shelf life [5]. However, the volatility and sensitivity of thyme oil present formulation challenges, highlighting the need for advanced encapsulation methods to maximize its utility. Among the various encapsulation strategies, the use of natural hydrocolloids as wall materials has emerged as a highly effective method [6]. These biopolymers are favored for their biocompatibility, versatility, and ability to form encapsulation matrices that can be finely tuned to modify the release behavior of the encapsulated compounds. Xanthan Gum, a high-molecular-weight polysaccharide produced by bacterial fermentation, is renowned for its exceptional thickening, stabilizing, and gel-forming properties [7]. Guar Gum, derived from the endosperm of the guar bean, is similarly valued for its thickening and stabilizing capabilities, with the added advantage of being highly soluble in cold water [8].

While the encapsulation of volatile compounds, such as essential oils, using natural hydrocolloids like Xanthan Gum [9] and Guar Gum [10] has been recognized for its potential in enhancing product stability and controlled release, there remains a significant research gap in understanding the nuanced interactions between these hydrocolloids and their collective impact on encapsulation dynamics. Most works focus on the impact on the rheological properties of both gums mixtures [11,12,13]. Despite the acknowledged individual benefits of Xanthan Gum [14] and Guar Gum [15] in encapsulation matrices—ranging from thickening and stabilizing to gel-forming capabilities—their synergistic effects, particularly in varying ratios, have not been thoroughly investigated for emulsion stability, encapsulation efficiency, and controlled release. This gap extends to a lack of clarity on how these interactions affect the encapsulation efficiency, stability, and release kinetics of volatile active compounds, such as essential thyme oil.

Furthermore, combining these hydrocolloids can potentially create synergistic effects, enhancing the encapsulation efficiency and modulating the release kinetics of encapsulated essential oils [16]. However, the impact of varying gum ratios on the encapsulation and release dynamics, especially for volatile compounds like essential thyme oil, remains insufficiently explored. Our study’s use of disc monoliths underscores the significant impact of encapsulation structure on the controlled release kinetics of volatile essential oils. These monoliths provide a uniform and structured medium that ensures consistent release behavior under specific environmental conditions. Their design facilitates accurate kinetic analysis by offering stable and uniform exposure to controlled temperature and airflow.

Modeling tools significantly advance the study of hydrocolloid interactions, particularly between Xanthan Gum and Guar Gum, in enhancing emulsion stability and enabling controlled release. The role of modeling in understanding hydrocolloid interaction was previously emphasized [17]. These tools provide deep insights into how gum ratios affect emulsion properties, enabling precise evaluation of hydrocolloid synergies on the encapsulation and release of active ingredients like essential oils. Through kinetic and diffusion analyses, modeling aids in optimizing encapsulation matrices for better stability and specific release patterns, streamlining formulation processes, and minimizing trial-and-error testing [18]. This underscores the importance of modeling in developing efficient encapsulation solutions across the food, pharmaceutical, and cosmetic sectors, marking a critical step forward in encapsulation technology research.

This study aims to bridge this gap by investigating essential thyme oil’s encapsulation and controlled release behavior using spray-dried powders with different Xanthan Gum and Guar Gum compositions. By employing kinetic modeling and analyzing effective diffusion coefficients, we seek to elucidate how the hydrocolloid composition influences the stability, encapsulation efficiency, and release kinetics of thyme oil volatiles. Through this research, we contribute to a broader understanding of hydrocolloid-based encapsulation systems, paving the way for developing tailored encapsulation strategies that optimize the functionality and application of essential oils in various industries.

## 2. Materials and Methods

### 2.1. Materials 

Essential thyme oil was purchased from Mundo dos Oleos (São Paulo, Brazil). Xanthan Gum and Guar gum were purchased from Adicel (São Paulo, Brazil). Oat cellulose fiber was purchased from Jelu-Werk (Rosenberg, Germany).

### 2.2. Production Methods 

#### 2.2.1. Emulsion Preparation 

Emulsions were prepared according to Table 1 and the method described in [16]. Increasing amounts of Xanthan Gum were combined with decreasing amounts of Guar Gum up to a concentration of both gums of 1 g/100 g. After complete solubilization of both gums, essential thyme oil at a ratio 1:1 (gum:oil) was added dropwise to the gum solution using stirring to form a coarse emulsion. Each coarse emulsion was stored at 5 °C, 25 °C, and 35 °C. After this step, each coarse emulsion was emulsified using an IKA TURRAX T-25 (IKA-Werke, Staufe, Germany) at 20,000 rpm for 2 min.

#### 2.2.2. Spray Drying 

Emulsions were spray dried using a lab-scale spray dryer (Labfirst Scientific Instruments, Shanghai, China) using a two-fluid nozzle (1 mm) in a co-current arrangement. The feed flow rate was set to 1 kg/h, the atomization drying gas flow rate was set to 1 kg/h, and the drying gas flow rate was set to 40 kg/h. The inlet–outlet temperatures were set to 200 °C/92 °C. Powder was collected after cyclone separation.

#### 2.2.3. Monoliths 

**Figure 1:**
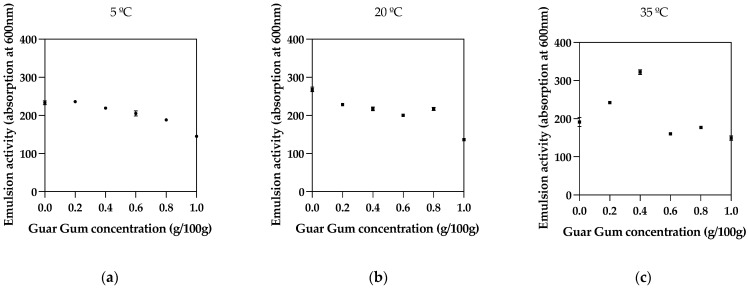
Emulsion activity for different Guar Gum concentrations and different temperatures: (**a**) 5 °C, (**b**) 20 °C, (**c**) 35 °C.

Cellulose-based disc monoliths were prepared by direct compression. Thus, 1 g of encapsulated oil powder was thoroughly mixed with 5 g of oat cellulose fiber and compressed with 20 tons for 2 min. The final disc dimensions are presented in Figure 1, with a diameter of 40 mm and a thickness of 5 mm.

### 2.3. Emulsion Characterization 

#### Emulsion Activity and Stability at Different Temperatures 

The emulsion activity was determined using a UV/Vis spectrophotometer (Model SP-220, Biospectro, Curitiba, PR, Brazil) with an absorbance function of 600 nm. After homogenization, the emulsion samples were diluted 1:100 by adding 1 mL of emulsion to 99 mL of distilled water [19,20]. The added water was at 5 °C, 20 °C, or 35 °C. The values obtained were directly used as emulsion activity. Homogenized samples were stored at three different temperatures, 5 °C, 20 °C, and 35 °C. After 24 h of the first measurement, subsequent absorbance measurements were taken to assess the emulsion stability. Emulsion stability values are determined by Equation (1).

(1) $$Emulsion\;Stability=\frac{A_{t}}{A_{0}}\times 100\%$$

where *A_t_*, is the absorbance measurement taken after *t* days of the emulsion homogenization, and *A*_0_ is the emulsion stability immediately after homogenization. 

### 2.4. Powder Characterization 

#### Process Drying Yield and Microencapsulation Efficiency 

Process yield was calculated by the ratio between the mass of powder collected, expressed in grams, with the total solids fed to the spray drying [21]. The latter is calculated by the fraction of emulsion solids multiplied by the total weight of each sample fed to the spray drying, as presented in Equation (2).

(2) $$Process\;Yield\;\left(\%\right)=\frac{Mass\;of\;powder\;collected\;in\;cyclone}{Total\;solid\;fraction\times Sample\;total\;weight\;}\times 100$$

The microencapsulation efficiency was determined according to [22]. This measurement indicates the essential oil’s ability to be retained by the encapsulating matrix and was determined based on the oil content and the oil content retained after the drying process. The percentage of essential thyme oil retained in the microcapsules was established according to a previously described methodology [23]. A sample weighing 100 mg was introduced into an ethanol–water mixture with a 1:1 ratio and agitated continuously overnight. The absorbance of the resultant solution was subsequently determined at a wavelength of 260 nm, employing carvacrol as the standard for comparison. Using a calibration curve, the concentration of the sample was calculated. The calibration curve was constructed based on various known concentrations of thyme essential oil, with absorbance readings taken at 260 nm. The encapsulation efficiency was calculated using Equation (3).

(3) $$EE\left(\%\right)=\frac{MEencap}{MEemulsion}\times 100$$

where *EE* is the encapsulation efficiency (%), *MEencap* is the mass of oil found in the powder (g), and *MEemulsion* is the mass of essential oil in the emulsion. 

### 2.5. Volatile Essential Oil Release Experiments and Kinetic Determination 

Disc-type monoliths of different encapsulated oils were placed in a drying oven (Solidsteel, Ribeirão Preto, Brazil) set at 35 °C and constant convective forced air. The oven size was 150 L, and air speed was 1.5 m/s. After reaching the desired temperatures, a sample was placed on a metallic tray, and weight measurements were taken each 30 min until the weight was constant for three consecutive measurements. For each sample, trials were repeated three times. 

### 2.6. Data Modeling 

#### 2.6.1. Kinetic Modeling

Zero- and first-order kinetic models were used to predict the property change of the different emulsions [24]. A basic equation describing the rate of change in the property can be written as Equation (4).

(4) $$-\frac{d\left(Pr\right)}{dt}=k{\left(Pr\right)}^{y}$$

where *Pr* is the property being studied, *k* is the rate constant of the property evolution, *y* is the order of the alteration reaction, and *t* is the time. For the zero-order equation (y = 0) and after integrating for time, Equation (5) is obtained:

(5) Pr=P0±kt

where *P*0 is the initial property level and *Pr* the emulsion stability at a given time *t*. For the first-order equation (y = 1) and after integrating for time, Equation (6) is obtained:

(6) Pr=P0×exp(±kt)

#### 2.6.2. Volatile Essential Oil Release Modeling

To determine the effective diffusion coefficient (*D*) from the first-order kinetic rate constants, we can use the relationship between the rate constant (*k*) and the diffusion coefficient. This relationship can be derived from Fick’s laws of diffusion, considering the geometry of the system and the boundary conditions. For a cylindrical disk where diffusion predominantly occurs through its thickness, the effective diffusion coefficient can be related to the first-order rate constant by Equation (7).

(7) $$D=\frac{kL^{2}}{\pi^{2}}$$

where *D* is the diffusion coefficient in m^2^/s, *k* is the first-order rate constant in s^−^^1^, and L is the half-thickness of the slab (for a disc, this would be half of the disc’s thickness). Alternatively, we can directly use a simplified version of Fick’s second law [25]. To apply Fick’s second law in a simplified form to your disc slab and estimate the diffusion coefficient D based on the given concentration profile over time, we consider the equation provided for the surface concentration Cs(t). This equation is particularly relevant for short times and can be simplified by truncating the series to the first term (*n* = 0) for an initial approximation. For the surface concentration Cs(t) at x = L/2, and limiting Fick’s terms to the first term, the simplified equation (Equation (8)) can be considered.

(8) $$C_{S}\left(t\right)=C_{0}\left[1-\frac{4}{\pi }\exp(-\frac{D\pi^{2}t}{L^{2}})\right]$$

where *C*_0_ is the initial concentration, *D* is the diffusion coefficient, *L* is the thickness of the slab, and t is the time. 

### 2.7. Modeling and Statistical Analysis 

All experiments were executed in triplicate, and the results were evaluated by one-way analysis of variance (ANOVA) and average comparison by Tukey’s test at 5% probability, using GraphPad Prism version 10.0.0 for MacOS GraphPad Software, Boston, MA, USA, www.graphpad.com. Modelling was performed using Python (version 3.12) (Python Software Foundation, Python Language Reference, available at https://www.python.org). Calculations were conducted using the scipy.optimize module and curve.fit function.

## 3. Results and Discussion

### 3.1. Emulsion Activity 

In this study, we evaluated the impact of varying concentrations of Guar Gum and Xanthan Gum on the emulsion properties of thyme oil emulsions across a thermal spectrum. The emulsions, differentiated by their respective ratios of Guar Gum to Xanthan Gum, and temperatures (5 °C, 20 °C, and 35 °C) were subjected to absorbance measurements at 600 nm to gauge their activity at each temperature. All emulsions were kept at each temperature before activity and stored at each temperature before stability measurements. Table 1 and Figure 1 present the resulting data, illustrating a distinct relationship between hydrocolloid composition and emulsion activity.

This study’s investigation into emulsion activity immediately after shearing reveals crucial insights into the role of hydrocolloid composition and shearing conditions in emulsion formation. After homogenizing the hydrocolloid mix with the oil at 20,000 rpm for 2 min, the emulsion’s stability is immediately assessed, capturing the initial interaction dynamics between the oil and hydrocolloids at various temperatures. In samples with a higher concentration of Guar Gum, such as Sample 1, the emulsion activity is robust across the temperature range, indicating that Guar Gum’s thickening properties effectively establish a stable emulsion post-shearing. This immediate stabilization is critical as it suggests that Guar Gum can rapidly form a cohesive network around oil droplets, preventing coalescence and separation. When examining samples with increasing Xanthan Gum content, we see that Sample 6, which solely contains Xanthan Gum, displays enhanced emulsion activity at higher temperatures. The rapid shearing process combined with Xanthan Gum’s unique properties likely enables the formation of a stable network resilient to thermal stress. This points to Xanthan Gum’s potential as a reliable stabilizer in applications where emulsions are exposed to higher temperatures.

The temperature at which shearing occurs plays a decisive role. While all samples exhibit significant emulsion activity at 5 °C, suggesting that more excellent conditions favor immediate emulsion stability, the response at higher temperatures varies. For instance, Sample 4, representing a balanced hydrocolloid mixture, achieves high emulsion activity at 35 °C. This indicates that combining Guar Gum and Xanthan Gum may provide a complementary effect, with each hydrocolloid’s properties synergizing to create an emulsion that is immediately stable after shearing and resilient to subsequent thermal challenges. The interactions between Xanthan Gum, Guar Gum, and carboxymethyl cellulose were previously reported, finding synergistic effects that influenced the flow behavior and droplet characteristics of oil-in-water emulsions, supporting the notion of complementary effects between Guar and Xanthan Gums [26].

Overall, the data imply that the formulation of hydrocolloids and the conditions under which they are sheared with the oil are instrumental in defining the emulsion’s immediate and possibly long-term stability. The ability of Guar Gum to establish primary stability and the thermal resilience imparted by Xanthan Gum highlight the importance of considering both composition and processing conditions in emulsion design. These findings offer a pathway to fine-tuning emulsion properties for various applications, emphasizing the need for a strategic approach to hydrocolloid selection and shearing parameters.

### 3.2. Emulsion Stability

The influence of varying concentrations of Guar Gum and Xanthan Gum on the stability of emulsions over three days under different temperature conditions was tested, and the results are presented in Table 1. On Day 1, a shift in stability patterns was noted. The emulsions demonstrated a general decrease in stability compared to Day 0, with the most pronounced stability observed in emulsions containing 1% Guar Gum, irrespective of the Xanthan Gum concentration. This indicates the strong stabilizing effect of Guar Gum when used alone. However, the stability was not uniform across all temperatures, suggesting that environmental conditions play a crucial role in the interaction dynamics between these hydrocolloids. By Day 2, the trend of decreasing stability halted. The observed increase in emulsion stability on Day 2, especially at 35 °C, may be attributed to the enhanced mobility of hydrocolloid molecules—Guar Gum and Xanthan Gum—due to elevated temperatures. This increased kinetic energy can facilitate reorganization at the molecular level, leading to stronger intermolecular interactions and a more robust emulsion matrix. Additionally, the heat may contribute to a reduction in the size of oil droplets, resulting from higher collision rates and improved homogenization, which enhances the emulsion’s stability. These changes, driven by thermodynamics and kinetics, suggest a system temporarily adapting to environmental conditions, potentially increasing stability through a more uniform droplet distribution and fortified hydrocolloid network. It was previously observed that the addition of Xanthan Gum and Guar Gum influenced the heat stability of oil-in-water emulsions, indicating that these hydrocolloids can affect the stability of emulsions under thermal conditions, likely through changes in the viscoelastic properties of the system [27].

Interestingly, the highest stability was again observed in samples with 1% Guar Gum at all temperatures. This reiterates the dominant role of Guar Gum in maintaining emulsion stability over time. The stability of emulsions with higher Xanthan Gum concentrations varied, indicating a complex interplay between hydrocolloid concentration and temperature. On Day 3, the stability of all emulsions had declined significantly. The sample with 0.4% Guar Gum and 0.6% Xanthan Gum exhibited the highest stability at 5 °C, while at 35 °C, the sample with 1% Xanthan Gum emerged as the most stable. This decline in stability across all samples highlights the temporal aspect of hydrocolloid-based emulsion systems.

Guar Gum and Xanthan Gum, known for their water-soluble characteristics, play a critical role in stabilizing emulsions. They enhance the viscosity of the aqueous phase, forming a viscoelastic network around oil droplets, thereby impeding coalescence. The initial stability observed, particularly in emulsions with higher Xanthan Gum concentrations, likely stems from a more structured and viscous continuous phase, which effectively immobilizes the oil droplets. On Day 0, the specific blend of 0.4% Guar Gum and 0.6% Xanthan Gum might have achieved an optimal viscosity–elasticity balance, contributing to the highest initial stability. However, as we observed over the subsequent days, several factors come into play, altering this stability.

Syneresis, or the gradual release of water from the polysaccharide network, might lead to a reduction in the continuous phase viscosity, consequently diminishing the emulsion’s stability [28]. Potential degradation or structural changes in polysaccharides, especially under higher temperatures, could weaken the network surrounding the oil droplets. The varying stability at different temperatures underscores the temperature-dependent behavior of these hydrocolloids [29]. With its unique helical structure, Xanthan Gum might form a more heat-resistant network than Guar Gum, explaining the relatively higher stability at elevated temperatures. Moreover, the phenomenon of Ostwald ripening, where larger droplets grow at the expense of smaller ones, could further contribute to the observed decrease in stability over time, particularly noticeable in emulsions with lower to moderate polysaccharide concentrations [30].

### 3.3. Kinetic Modeling of Emulsion Stability

**Figure 2:**
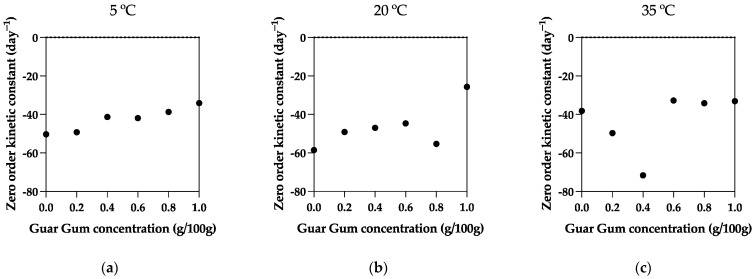
Emulsion stability zero-order kinetic constants (**a**) 5 °C, (**b**) 20 °C, and (**c**) 35 °C.

In our study on emulsion systems comprising Guar Gum and Xanthan Gum, kinetic modeling was employed to dissect the stability dynamics across various samples and temperatures. Applying zero-order and first-order kinetic models has shed light on the intricate molecular interactions dictating the stability of these emulsions. Figure 2 presents the zero-order kinetic constants against Guar Gum concentration, while Table 2 presents the kinetic modeling made with both zero- and first-order models.

An average R^2^ value of 0.891 for the zero-order kinetic model suggests that the rate of emulsion stability loss is consistent over time, irrespective of the concentration of the emulsified phase. This level of fit implies that external factors, rather than the dispersed phase concentration, may be driving the loss of stability. Such factors could include constant environmental conditions, like temperature or pH, that persistently affect the emulsion’s stability.

The implication here is that the destabilizing process, whether due to coalescence, flocculation, or creaming, is occurring steadily. Similarly, the average R^2^ value of 0.893 for the first-order kinetic model indicates that the emulsion stability loss is also well described by a process proportional to the emulsion’s dispersed phase concentration. This suggests that the rate of destabilization slows down as the emulsion becomes less stable, which could reflect a range of phenomena, including the depletion of emulsifiers at the droplet interface or a decrease in the rate of droplet collision as the system becomes more coalesced and the remaining droplets are further apart. The similar R^2^ values for both zero-order and first-order kinetics suggest that the loss of emulsion stability may involve a combination of factors that align with both models. For instance, certain stages of the emulsion’s life may be better described by zero-order kinetics (e.g., when a saturation of surfactant molecules at the interface occurs), while other stages may transition to first-order behavior as the concentration of intact droplets decreases.

The kinetic constants for emulsion stability, especially at intermediate concentrations of Guar Gum and Xanthan Gum, exhibit intriguing non-linear behavior that deviates from the expectations of the Arrhenius theory. Typically, the Arrhenius theory predicts a smooth increase in reaction rates with temperature due to more molecules having sufficient energy to overcome the activation energy barrier. However, the observed data show a peculiar trend at 20 °C, with intermediate hydrocolloid concentrations experiencing a peak in degradation rates for both zero-order and first-order kinetics. This could be related to the potential synergistic effects of both molecules, especially at 20 °C [26]. It was previously found that Xanthan Gum at 1.0 g/100 g also did not abide by the Arrhenius equation [31].

This anomalous trend implies that the interactions between Guar Gum and Xanthan Gum are more complex than previously thought, particularly when it comes to the emulsion’s response to temperature. At 20 °C, the unique combination of these hydrocolloids may form a matrix that is susceptible to destabilization, possibly due to a particular balance of molecular forces at this temperature that does not hold at lower or higher temperatures. The temperature might induce changes in the hydration levels or the conformation of the polysaccharide chains within the hydrocolloids, leading to a less stable emulsion structure that is prone to breakdown. Moreover, the specific temperature of 20 °C could correspond to a phase transition within the emulsion system that is more pronounced at these intermediate concentrations. Such a transition could impact the viscosity or solubility of the hydrocolloids and consequently affect the stability of the emulsion. The synergistic interaction between Xanthan and Guar Gum was previously discussed and suggests that gelation and transition temperatures of dynamic viscoelasticity for mixtures with native xanthan were observed at 25 and 30 °C, respectively [32]. This could indicate that a phase transition in the emulsion system may occur around these temperatures, which might impact the stability of the emulsion. Additionally, the rate of stability loss may be influenced by a switch in the dominant mechanism of breakdown, such as Ostwald ripening or coalescence, which could be facilitated by the hydrocolloid ratios at this intermediate temperature.

The non-linearity and deviation from the expected Arrhenius behavior highlight the complexity of the emulsion system and the significant role of hydrocolloid concentration and temperature in determining stability. These findings are crucial for the formulation of stable emulsions, suggesting that a product’s exposure to a specific temperature range must be carefully considered to ensure quality and performance. Understanding these dynamic interactions is key to advancing emulsion technology and developing products that maintain their stability under diverse conditions.

### 3.4. Encapsulation of Thyme Oil Emulsions Via Spray Drying

Table 3 presents the results from spray-drying powder characterization. The process yield from spray drying varied subtly across the samples, influenced significantly by the composition of Guar Gum and Xanthan Gum. Notably, samples with a balanced mix, such as Sample 3 (0.6 g/100 g Guar Gum and 0.4 g/100 g Xanthan Gum) and Sample 4 (0.4 g/100 g Guar Gum and 0.6 g/100 g Xanthan Gum), demonstrated marginally higher yields above 70%. The observed variations in drying yield across samples containing different ratios of Guar Gum (GG) and Xanthan Gum (XG) can be attributed to the distinct viscosities imparted by each hydrocolloid blend, affecting the atomization process during spray drying. It is hypothesized that certain GG and XG combinations may optimize the emulsion’s viscosity in a manner that is conducive to efficient droplet formation when subjected to the shear forces within the nozzle. This optimized droplet formation is characterized by a narrower size dispersion, which is a crucial factor in the drying process [33].

Higher yields do not necessarily indicate more efficient drying kinetics but may instead reflect the cyclone’s efficiency in particle recovery. While cyclones are less effective at capturing very small particles due to inertia, they are also less efficient at capturing larger, stickier particles, which may adhere to the drying chamber walls due to a lower glass transition temperature (Tg). Stickiness, often exacerbated by higher moisture content, can lead to a lower Tg and cause particles to adhere to the chamber walls [34]. This adhesion reduces the recovery yield, whereas an optimized hydrocolloid blend can help mitigate this effect by enhancing drying kinetics, thereby reducing moisture content and potential stickiness.

Therefore, the process yield and encapsulation efficiency results must be considered in the context of particle size distribution and the physical properties of the particles post-drying. It is the interplay between the hydrocolloid-induced viscosity, particle size, and drying conditions that defines the overall efficiency of the spray-drying process [35]. Further investigation into the relationship between hydrocolloid ratios, viscosity, and particle behavior during spray drying is necessary to elucidate these complex dynamics fully. Conversely, the presence of Guar Gum alone or in predominant ratios slightly decreased the yields, hinting at the individual gum properties affecting either the drying efficiency or the recovery of the dried product.

The encapsulation efficiency, a crucial parameter measuring the retention of essential thyme oil within the spray-dried particles, showed a marked improvement with increasing Xanthan Gum proportions. The highest encapsulation efficiency was observed in Sample 4, where Xanthan Gum was more prevalent. This pattern indicates that Xanthan Gum’s molecular structure, possibly due to its branched configuration, provides a more effective barrier against oil loss during the drying process. The encapsulation efficiency’s enhancement with Xanthan Gum underscores its suitability in formulations aimed at the retention of volatile compounds through spray drying. Similar to what was previously found, samples with higher emulsion activity resulted in higher encapsulation effectiveness [36].

### 3.5. Release of Thyme Essential Oil from the Encapsulated Material

**Figure 3:**
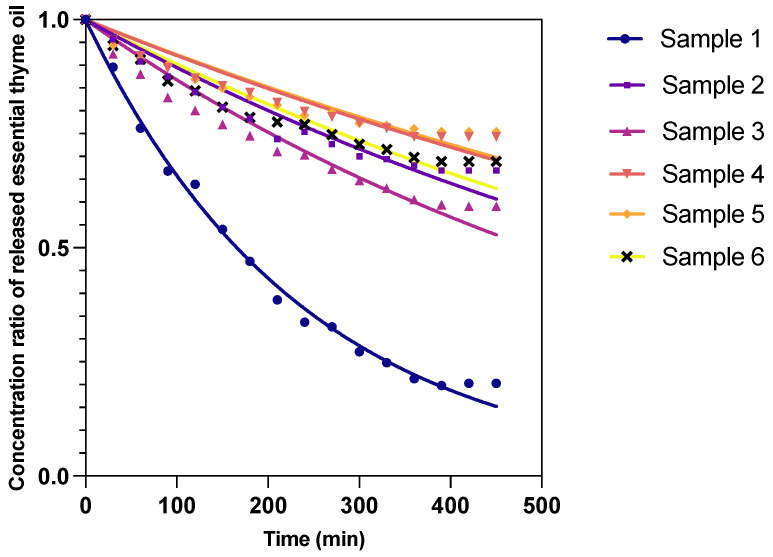
Release profile of encapsulated essential thyme oil.

The controlled release of thyme oil volatiles from encapsulated powders, subjected to a steady environment in a convective oven at 35 °C, was determined. The normalized volatile weight loss across six samples is presented in Figure 3. We analyzed the effect of varying gum compositions on the release dynamics over time. Our encapsulation approach utilized monoliths composed of 5 g of cellulose fiber and 1 g of encapsulation powder, derived from different ratios of Guar Gum and Xanthan Gum.

During the initial 150 min, a gradual decline in the oil content ratio was observed across all samples, indicative of the onset of volatile release. Sample 1, characterized by a higher concentration of Guar Gum and devoid of Xanthan Gum, demonstrated a more rapid release, with the oil content ratio decreasing to 0.540. This contrasts with Sample 4, where a higher Xanthan Gum concentration appeared to slow the release, maintaining a higher oil content ratio of 0.855. The differential release rates in this early phase suggest that Xanthan Gum’s structural properties contribute to a more controlled release mechanism, potentially due to its ability to form a denser matrix that restricts volatile escape [7].

As the experiment progressed to the 150–300 min window, the decline in oil content ratios continued, albeit at a more moderated pace. This stage highlighted the sustained release capabilities of the encapsulation matrices, with Sample 4 consistently exhibiting slower release rates compared to Sample 1. The encapsulation efficiency and the structural integrity afforded by the hydrocolloid mixtures are crucial in achieving a controlled release profile, with the Xanthan Gum-rich samples showcasing an enhanced ability to retain volatiles longer. Towards the later stages of the observation period, the oil content ratios across samples began to approach a plateau, indicating a near-equilibrium state. In a previous work, a volatile release plateau was also identified by [37]. Remarkably, from 270 min onwards, the changes in oil content ratios became minimal, suggesting that most readily releasable volatiles had been exhausted. Notably, Samples 2 through 6 showed a convergence in release behavior, illustrating a common threshold beyond which the gum composition’s impact on release rate diminishes. Sample 1, with its unique Guar Gum composition, stabilized at a slightly distinct plateau, further emphasizing the nuanced influence of gum ratios on the encapsulation and release phenomena.

This comprehensive analysis reveals that the encapsulation and subsequent release of thyme oil volatiles are intricately linked to the hydrocolloid composition of the encapsulation matrix. The slower release rates observed in samples with higher Xanthan Gum content underscore the gum’s structural benefits in creating a robust encapsulation matrix. Conversely, the quicker release observed in Guar Gum-dominant samples points to a less restrictive matrix, facilitating the faster release of volatiles [38]. The eventual stabilization of release rates underscores a balance between the volatiles’ diffusion out of the matrix and the diminishing concentration gradient. The nuanced understanding garnered from this study highlights the potential of leveraging hydrocolloid properties to tailor the release profiles of encapsulated volatiles. By manipulating the ratios of Guar Gum and Xanthan Gum, it is possible to design encapsulation systems with desired release kinetics suitable for various applications, where the controlled release of active compounds is crucial [39].

### 3.6. Kinetic Modeling of Thyme Oil Release and Effective Diffusivity

Table 4 presents the results from the first-order kinetic modeling and the determination of diffusivity using two methods.

Sample 1, with an exclusive Guar Gum formulation, demonstrated a notably higher release rate (k = 1.46 *×* 10*^−^*^10^ min*^−^*^1^) and a more substantial effective diffusion coefficient (D_eff_ = 2.4 *×* 10*^−^*^10^ m^2^/s). Due to its linear molecular structure, this suggests that Guar Gum may facilitate a less restrictive matrix, allowing for a more rapid oil diffusion. In a previous study, Guar Gum surface modification led to higher retention ability due to increased steric hinderance [40]. This sample’s relatively higher D_eff_ value points towards a matrix that permits greater mobility of oil molecules, potentially leading to quicker release rates. Conversely, samples with increasing Xanthan Gum content tended toward slower release rates and lower D_eff_ values. For instance, Sample 4, which contains a higher proportion of Xanthan Gum (0.6 GX to 0.4 GG), showed one of the lowest D_eff_ values (0.7 *×* 10*^−^*^10^ m^2^/s) among the samples. This aligns with the understanding that Xanthan Gum’s branched structure creates a denser and more complex network within the encapsulation matrix. Such a network likely acts as a barrier to the mobility of oil molecules, resulting in a decelerated diffusion process. This pattern underscores the critical impact of hydrocolloid composition on the release dynamics of encapsulated thyme oil. The interplay between Guar Gum and Xanthan Gum within the encapsulation matrix is decisive in determining the diffusion characteristics of the oil. While Guar Gum seems to promote a quicker release, Xanthan Gum appears to slow down the process, highlighting the balance that must be achieved between these hydrocolloids to tailor the encapsulation system to specific release requirements.

In examining the diffusion behavior of thyme oil through the encapsulation matrix, our study utilized two distinct methodologies to calculate the effective diffusion coefficients (D_eff_), one derived from the kinetic rate constant and the other based on Fick’s second law of diffusion. A comparison between these two calculated D_eff_ values shed light on the intricate dynamics of thyme oil release from hydrocolloid matrices. For instance, Sample 1, characterized by a Guar Gum-exclusive formulation, showcased a D_eff_ value of 2.4 *×* 10*^−^*^10^ m^2^/s when derived from the kinetic rate constant. This value, when juxtaposed with the D_eff_ calculated using Fick’s second law (9.8 *×* 10*^−^*^10^ m^2^/s), reveals a discrepancy that may be attributed to the different assumptions and methodologies inherent to each calculation approach. The kinetic rate constant-based calculation assumes a homogeneous release mechanism across the matrix, which might oversimplify the complex interactions between thyme oil molecules and the hydrocolloid network. On the other hand, the application of Fick’s second law considers the spatial concentration gradients within the matrix, offering a more nuanced view of diffusion that considers the heterogeneity of the system [41].

As the concentration of Xanthan Gum increases in the formulations, we observe a consistent trend where D_eff_ values derived from kinetic constants tend to be lower than those calculated via Fick’s second law. The increase in Xanthan Gum concentration within the emulsion formulations consistently results in lower effective diffusivity (D_eff_) values derived from kinetic constants compared to those calculated using Fick’s second law. This trend suggests that the release from these emulsions is governed by more than just diffusion; the gel-like nature of Xanthan Gum likely creates a more resistant matrix, affecting overall release kinetics. The fact that the kinetic-derived D_eff_ is substantially lower points to the presence of complex interactions and mass transfer phenomena within the hydrocolloid matrix that are intensified with higher Xanthan Gum levels, ultimately influencing the controlled release behavior of the encapsulated oils.

This observation could indicate that the release of thyme oil from matrices with higher Xanthan Gum content is more significantly impacted by the concentration gradients within the matrix, which are more accurately captured by Fick’s second law. The difference in D_eff_ values calculated using the two approaches emphasizes the role of hydrocolloid composition in influencing the diffusion and release mechanisms of encapsulated substances.

### 3.7. Correlation Analysis of Different Modeling Parameters

**Figure 4:**
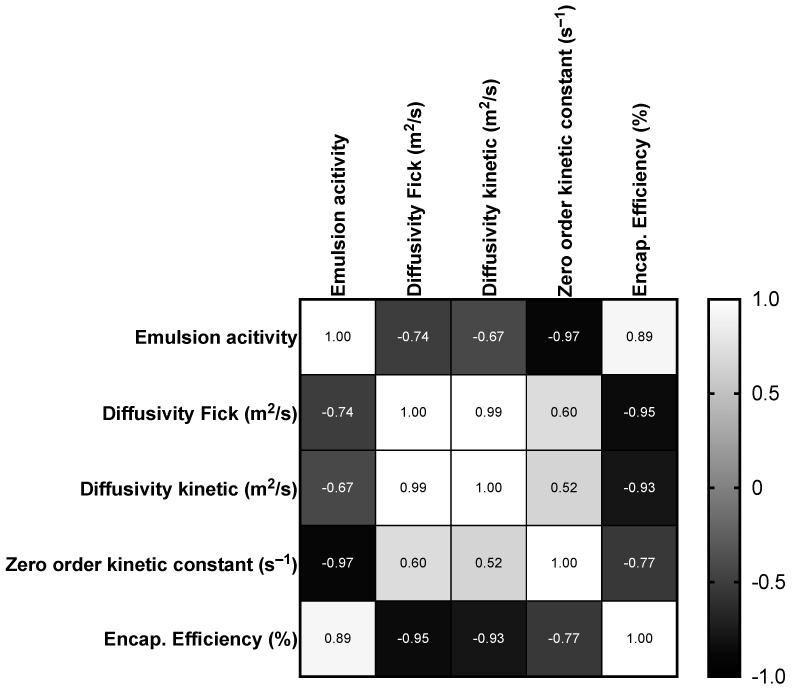
Pearson correlation matrix of emulsion characteristics.

The correlation matrix in Figure 4 indicates distinct relationships between hydrocolloid composition and the key parameters of emulsion systems. Specifically, it sheds light on the effects of Guar Gum and Xanthan Gum on emulsion activity, diffusivity measured by two methods (Fick’s law and kinetic analysis), kinetic stability constants, and encapsulation efficiency. A pronounced negative correlation between emulsion activity and the zero-order kinetic constant (−0.972) suggests that emulsions exhibiting higher initial activity, potentially due to more synergistic hydrocolloid interactions, tend to have a slower rate of stability degradation. This finding implies that an optimal balance of Guar Gum and Xanthan Gum contributes to a more stable emulsion that retains its structural integrity over time. The positive correlation between emulsion activity and encapsulation efficiency (0.886) further supports this, indicating that proper hydrocolloid composition is crucial for enhancing the emulsion’s ability to effectively encapsulate and retain the oil.

Furthermore, the negative correlation between emulsion activity and both measures of diffusivity, Fick (−0.740) and kinetic (−0.673), highlights that higher initial activity is generally accompanied by a lower rate of molecule movement within the emulsion matrix. The substantial positive correlation between the two diffusivity measurements (0.995) confirms that both capture a consistent aspect of molecule mobility, despite being derived from different theoretical frameworks. The strong negative correlations of Fick diffusivity (−0.955) and kinetic diffusivity (−0.927) with encapsulation efficiency underscore the inverse relationship between the rate of molecule movement through the matrix and the emulsion’s ability to maintain encapsulated substance integrity. In a previous work, the authors found that an increasing wall material concentration, and, thus, less molecule movement, increases encapsulation efficiency [42]. Essentially, the denser and more complex the hydrocolloid network—likely with higher Xanthan Gum content—the slower the diffusivity and the higher the encapsulation efficiency, indicating a more controlled release of the encapsulated compound due to increased molecular interaction between components [39].

Guar Gum and Xanthan Gum’s interactions within the emulsion matrix significantly influence not just the initial activity and stability but also the movement of encapsulated oils and the effectiveness of their encapsulation. This analysis underpins the importance of hydrocolloid ratio precision in emulsion formulation, providing the necessary insight to engineer emulsions with specific release rates, stability profiles, and encapsulation efficiencies tailored to meet the demands of various industry applications.

## 4. Conclusions

In conclusion, this study highlighted the critical influence of hydrocolloid composition—specifically the ratio of Guar Gum to Xanthan Gum—on the stability, diffusivity, and encapsulation efficiency of thyme oil emulsions. Our findings underscore that a nuanced balance between Guar Gum and Xanthan Gum is essential for optimizing emulsion characteristics. Guar Gum’s structure was associated with increased diffusivity and faster stability degradation, whereas Xanthan Gum contributed to reduced diffusivity, improved stability, and enhanced encapsulation efficiency due to its denser matrix formation. The observed correlations between hydrocolloid interactions and emulsion properties emphasize the complexity of formulating emulsion-based delivery systems. This research provides foundational insights for tailoring emulsion formulations to achieve desired outcomes, such as controlled release rates and prolonged stability, which are crucial for their application across various industries. Future work will further elucidate the underlying mechanisms of hydrocolloid interactions, guiding the development of innovative encapsulation technologies.

## Figures and Tables

**Table 1:** Gum formulation used in essential thyme oil emulsion, emulsion activity, and emulsion stability.

	Guar Gum Concentration (g/100 g)	Xanthan Gum Concentration (g/100 g)	Temperature (°C)	Emulsion Activity	Emulsion Stability		
				Day 0 (abs 600 nm)	Day 1 (%)	Day 2 (%)	Day 3 (%)
Sample 1	1.0	0.0	5	145.1 ± 0.82	95 ± 1.7	73 ± 3.74	33 ± 4.9
			20	126 ± 2.45	86 ± 4.5	90 ± 3.3	33 ± 1.7
			35	133 ± 5.79	80 ± 1.4	94 ± 1.7	22 ± 2.6
Sample 2	0.8	0.2	5	180 ± 3.30	72.5 ± 0.82	84 ± 6.02	33 ± 4.3
			20	215 ± 2.45	62.3 ± 1.63	71.6 ± 0.82	17.2 ± 0.87
			35	166 ± 3.56	75 ± 2.05	91 ± 2.6	36 ± 2.83
Sample 3	0.6	0.4	5	213 ± 6.94	66 ± 1.25	65 ± 1.7	34 ± 2.94
			20	200 ± 1.89	78 ± 1.63	71 ± 1.25	28 ± 1.25
			35	145 ± 3.09	84 ± 2.05	94 ± 1.7	28 ± 0.93
Sample 4	0.4	0.6	5	231 ± 3.30	66 ± 3.68	64 ± 2.62	41 ± 2.76
			20	217 ± 4.97	72 ± 3.77	62 ± 1.41	31 ± 2.05
			35	322 ± 6.38	53 ± 2.49	57 ± 2.94	27 ± 1.4
Sample 5	0.2	0.8	5	236 ± 2.16	66.5 ± 0.82	53 ± 4.03	34 ± 3.59
			20	228 ± 3.09	68 ± 2.45	65 ± 1.89	29 ± 4.3
			35	242 ± 3.56	64 ± 2.05	68 ± 2.87	32.9 ± 0.47
Sample 6	0	1.0	5	233 ± 4.71	72 ± 0.82	48 ± 2.16	36 ±1.25
			20	268 ± 6.02	59.7 ± 0.94	66 ± 1.89	29 ± 1.1
			35	191 ± 11.9	75.4 ± 0.94	91 ± 1.7	38 ± 1.4

**Table 2:** Zero- and first-order kinetic modeling of the emulsion stability over different ranges of Guar and Xanthan Gum and temperatures.

	Temperature (°C)	Zero Order Kinetic Model			First Order Kinetic Model		
		Y-Intercept	Slope (Day^−1^)	R Squared	Y-Intercept	Slope (Day^−1^)	R Squared
Sample 1	5	147 ± 2.8	−34 ± 1.5	0.98	153 ± 5.8	−0.35 ± 0.03	0.98
	20	136.0 ± 8.7	−25 ± 4.6	0.75	135 ± 23.4	−0.23 ± 0.12	0.87
	35	149 ± 10.1	−33 ± 5.4	0.78	146 ± 29	−0.28 ± 0.15	0.88
Sample 2	5	188 ± 6.8	−38 ± 3.6	0.91	189 ± 21.5	−0.27 ± 0.09	0.85
	20	218 ± 8.5	−55 ± 4.5	0.93	221 ± 29.7	−0.38 ± 0.13	0.87
	35	177 ± 7.1	−34 ± 3.8	0.91	177 ± 21.6	−0.24 ± 0.09	0.81
Sample 3	5	205 ± 8.3	−41 ± 4.5	0.89	210 ± 20.6	−0.29 ± 0.08	0.89
	20	206.8 ± 8.5	−44 ± 4.5	0.91	207 ± 26.4	−0.29 ± 0.10	0.84
	35	156 ± 10.4	−29 ± 5.6	0.64	158 ± 29.0	−0.26 ± 0.14	0.77
Sample 4	5	219 ± 8.3	−41 ± 4.5	0.89	225 ± 18.5	−0.26 ± 0.06	0.91
	20	214 ± 5.4	−47 ± 2.9	0.96	219 ± 17.2	−0.32 ± 0.06	0.94
	35	298 ± 13.9	−71 ± 7.4	0.90	314 ± 28.6	−0.39 ± 0.08	0.93
Sample 5	5	224 ± 6.3	−49 ± 3.4	0.95	233 ± 9.8	−0.34 ± 0.04	0.98
	20	224 ± 8.5	−49 ± 4.5	0.92	228 ± 23.7	−0.32 ± 0.08	0.89
	35	235 ± 7.9	−49 ± 4.2	0.93	241 ± 20.9	−0.31 ± 0.07	0.92
Sample 6	5	225 ± 4.8	−50 ± 2.5	0.97	234 ± 5	−0.34 ± 0.02	0.99
	20	259 ± 8.1	−58 ± 4.3	0.94	267 ± 21.3	−0.34 ± 0.06	0.94
	35	204 ± 7.9	−38 ± 4.2	0.88	203 ± 23.9	−0.24 ± 0.08	0.82

**Table 3:** Results of process yield and encapsulation efficiency across different samples.

Sample	Process Yield (g/100 g)	Encapsulation Efficiency (g/100 g)
1	74 ± 4.3	36.3 ± 0.31
2	72 ± 7.2	59.5 ± 0.75
3	77 ± 5.3	50.3 ± 0.42
4	78 ± 3.1	74.4 ± 0.63
5	75 ± 3.8	69.3 ± 0.46
6	75 ± 5.7	62.0 ± 0.53

**Table 4:** First-order kinetic and Fick’s second law modeling of essential oil thyme release at 35 °C.

	M_0_ (g)	K (min^−1^)	D_k_ (×10^−10^ m^2^/s)	R^2^	D_eff_ (×10^−10^ m^2^/s)	R^2^
Sample 1	0.3456 ± 0.0139	1.46 × 10^−3^ ± 1.88 × 10^−4^	9.8 ± 0.32	0.9541	2.4 ± 0.26	0.9928
Sample 2	0.5746 ± 0.0136	6.69 × 10^−4^ ± 9.82 × 10^−5^	4.5 ± 0.15	0.9389	1.1 ± 0.15	0.9125
Sample 3	0.4817 ± 0.0104	7.75 × 10^−4^ ± 9.05 × 10^−5^	5.2 ± 0.21	0.9606	1.3 ± 0.17	0.9321
Sample 4	0.7194 ± 0.0125	4.16 × 10^−4^ ± 7.07 × 10^−5^	3.5 ± 0.13	0.9463	0.7 ± 0.012	0.8935
Sample 5	0.6652 ± 0.0135	4.83 × 10^−4^ ± 8.25 × 10^−5^	3.4 ± 0.15	0.9186	0.82 ± 0.014	0.8234
Sample 6	0.5952 ± 0.0129	6.06 × 10^−4^ ± 8.92 × 10^−5^	4.1 ± 0.52	0.9385	1.0 ± 0.15	0.8852

M_0_ initial concentration, k—first order kinetic constant, D—diffusion coefficient determined from kinetic constant, D_eff_—effective diffusion coefficient determine from Fick’s second law (*n* = 0).

## Data Availability

The original contributions presented in the study are included in the article, further inquiries can be directed to the corresponding author.
